# Effectiveness of Parent Education in Pivotal Response Treatment on Pivotal and Collateral Responses

**DOI:** 10.1007/s10803-019-04061-6

**Published:** 2019-05-24

**Authors:** Rianne Verschuur, Bibi Huskens, Robert Didden

**Affiliations:** 1 Behavioural Science Institute, Radboud University, Montessorilaan 3, 6525 HR Nijmegen, The Netherlands; 2 Dr. Leo Kannerhuis, Department of Research, Development & Innovation, Ireneweg 16, 6882 HL Oosterbeek, The Netherlands; 3 Seyscentra, De Horst 6, 6581 TE Malden, The Netherlands

**Keywords:** Autism spectrum disorder, Pivotal response treatment, Parent education, Parent-created opportunities, Child initiations

## Abstract

In two studies, we investigated the effectiveness of parent education in Pivotal Response Treatment (PRT) on parent-created opportunities and spontaneous child initiations in two community-based treatment facilities for children with autism spectrum disorder (ASD). Changes in parental stress and self-efficacy were explored. Participants were 26 parents and their children who participated in group (Study 1) or individual (Study 2) parent education in PRT. Results indicated that group-based parent education resulted in moderate increases in opportunities, functional initiations, and empathic social initiations. Furthermore, parental stress reduced and self-efficacy increased. Individual parent education resulted in large increases in opportunities and functional initiations, but parental stress and self-efficacy did not change. Implications for clinical practice and directions for future research are discussed.

**Electronic supplementary material:**

The online version of this article (10.1007/s10803-019-04061-6) contains supplementary material, which is available to authorized users.

## Introduction

Parenting a child with autism spectrum disorder (ASD) is more demanding than parenting a typically developing child or a child with other developmental disabilities (Hayes and Watson 2013). ASD is a neurodevelopmental disorder characterized by impairments in social communication and social interaction, and restricted, repetitive, and stereotyped behaviors (American Psychiatric Association 2013). Many children with ASD also exhibit challenging behaviors such as tantrums, aggression, and self-injurious behavior (Jang et al. 2011; Matson et al. 2009). Furthermore, approximately 70–92% of the children with ASD meet criteria for at least one comorbid psychiatric diagnosis, including attention deficit hyperactivity disorder (ADHD), oppositional defiant disorder (ODD), anxiety disorders, and mood disorders (Brookman-Frazee et al. 2018; Joshi et al. 2010; Simonoff et al. 2008). The majority of children with ASD have intelligence quotient (IQ) scores above 70 and are thus not classified as having an intellectual disability (Baio et al. 2018; Joshi et al. 2014). The characteristics of ASD and associated challenging behaviors and comorbid psychopathology not only impact children with ASD but also their parents (Karst and Van Hecke 2012). Parents of children with ASD report more parental stress, lower levels of parental self-efficacy, and less overall well-being (e.g., Frantz et al. 2018; Hayes and Watson 2013; Karst and van Hecke 2012). Furthermore, researchers have identified higher prevalence of depression and anxiety among parents of children with ASD (e.g., Bitsika and Sharpley 2004; Frantz et al. 2018; Singer 2006). When the severity of ASD symptoms, challenging behaviors, or comorbid psychopathology exceed the ability of parents to cope, the likelihood of psychiatric hospitalization or inpatient treatment increases (Mandell et al. 2012; Righi et al. 2018). Indeed, approximately 6% of children with ASD receive inpatient treatment (Cidav et al. 2013).

In early childhood, children with ASD engage in fewer initiations than typically developing children and their initiations serve fewer functions (Stone et al. 1997; Wetherby and Prutting 1984). These deficits in initiations continue beyond early childhood. School-aged children, adolescents, and adults with ASD initiate social conversation less often which may interfere with the development of social relationships (Hauck et al. 1995; Koegel et al. 2016b; Stone and Caro-Martinez 1990). In addition, deficits in initiations often lead to directive parent behaviors because parents tend to compensate for their child’s lack of initiations rather than providing him or her with opportunities to initiate (Hudry et al. 2013; Wan et al. 2012). Children’s challenging behaviors might also contribute to directive parent behavior and further reduce children’s opportunities to initiate (Reed and Osborne 2014; Shawler and Sullivan 2017).

Parent education has long been accepted as a beneficial method to teach parents skills to improve their child’s skills and reduce their child’s challenging behaviors (e.g., McConachie and Diggle 2007; Steiner et al. 2012). As a collateral result, reductions in parental stress and increases in parental self-efficacy may occur (Brookman-Frazee et al. 2009; Da Paz and Wallander 2017; Steiner et al. 2012). Parent education also increases intervention intensity because parents are able to provide intervention throughout the day and in various natural settings, increasing the child’s rate of progress and promoting generalized use of skills (Steiner et al. 2012). In addition, due to the increase in the number of children diagnosed with ASD, parent education is necessary to meet increased demands for treatment services (Elsabbagh et al. 2012; Steiner et al. 2012).

Parent education is an essential component of Pivotal Response Treatment (PRT; Koegel et al. 2016a). PRT is a naturalistic evidence-based intervention that targets pivotal skills (e.g., initiations) in children with ASD to produce generalized improvements across domains of functioning using the principles of Applied Behavior Analysis (ABA). A systematic review has reported evidence for the effectiveness of PRT for increasing initiations and producing collateral improvements in communication, language, affect, play, and challenging behaviors (Verschuur et al. 2014). Although the effectiveness of PRT has mainly been demonstrated in preschool children with ASD and cognitive impairments, some studies indicate that school-aged children with average cognitive abilities may also benefit from PRT (e.g., Doggett et al. 2013; Huskens et al. 2012; Verschuur et al. 2017).

A large number of studies on PRT focused on parent education as PRT is designed to be conducted in natural environments (e.g., Bradshaw et al. 2017; Coolican et al. 2010; Hardan et al. 2015; Nefdt et al. 2010; Randolph et al. 2011). Parents are taught to create opportunities for their child to initiate individually, in a group, or through a self-directed learning program. Results of studies on parent education in PRT suggest that parents can be taught to implement PRT. Most parents meet criteria for fidelity of PRT implementation after parent education (e.g., Verschuur et al. 2014). However, in most studies, parent education in PRT was conducted by trainers who were employed by university-based research clinics. An exception is the study by Bryson et al. (2007) in which community service providers were taught to educate parents in PRT, but this study did not use an experimental design and presented only preliminary findings. To allow for dissemination of PRT to a large number of children it is important that parent education can be effectively delivered by PRT trainers who are part of community-based treatment facilities (e.g., Brookman-Frazee et al. 2012b; Bryson et al. 2007) and thus more research on this topic is warranted. In addition, it is important to determine the effectiveness of parent education in PRT for parents of school-aged children with ASD, average cognitive abilities, and comorbid psychopathology, as there is limited information about the effectiveness of PRT in this population (e.g., Verschuur et al. 2017). Furthermore, thus far there is little evidence for collateral changes in parental stress and parental self-efficacy as a result of parent education in PRT (Verschuur et al. 2014). Finally, additional research on the effectiveness of group parent education in PRT is warranted, because only a couple of studies using a group model were conducted (e.g. Bryson et al. 2007; Gengoux et al. 2015; Hardan et al. 2015; Minjarez et al. 2011, 2013). Although these studies reported improvements in parent and child behaviors, conclusive evidence for the effectiveness of group-based parent education in PRT is still limited, as Bryson et al. (2007) and Minjarez et al. (2011, 2013) did not use an experimental design.

To address these needs, the objective of the present study was to investigate the effectiveness of parent education in PRT on parent-created opportunities and child initiations in two community-based treatment facilities for children with ASD in the Netherlands. Furthermore, collateral changes in parental stress and parental self-efficacy were explored. To this end, we conducted two separate single-case design studies of parent education in PRT. In Study 1, the effectiveness of a group parent education program was evaluated. In Study 2, we investigated the effectiveness of individual parent education.

## Study 1: Group Parent Education in PRT

### Method

#### Setting and Participants

Study 1 was conducted at the Dr. Leo Kannerhuis, which is a specialized treatment center for children with ASD in the Netherlands. Children received outpatient treatment, day treatment, or inpatient treatment because of severe autism symptoms, comorbid psychopathology, challenging behaviors, and/or difficulties in family functioning. Child psychologists and child psychiatrists referred potential participants to the study. Children were included if they met the following inclusion criteria: (a) clinical diagnosis of ASD according to the DSM-IV-TR or DSM-5 criteria (American Psychiatric Association 2000, 2013) and confirmed by scores on the Social Responsiveness Scale (SRS-2; Constantino and Gruber 2012; Dutch version by Roeyers et al. 2015) and/or Autism Diagnostic Observation Schedule (ADOS; Lord et al. 2012; Dutch version by De Bildt et al. 2013), (b) aged between 3 and 15 years at baseline, and (c) total IQ, verbal/reasoning IQ, or performance IQ above 70 on the Dutch version of the Wechsler Intelligence Scale for Children-III (WISC-III-NL; Kort et al. 2005) or the Snijders-Oomen nonverbal intelligence scale-revised 2½-7 (SON-R 2½-7; Tellegen et al. 1998). Only children with IQ scores above 70 were included because the specialized treatment center did not provide treatment to children with intellectual disabilities. Additionally, if a child received inpatient treatment during the period of data collection, the child and his or her parents were required to meet at least once every week at home or at the treatment facility. Children were not excluded if they received interventions in addition to PRT (e.g., milieu therapy, speech-language therapy, art therapy, family therapy, pharmacological interventions, or physiotherapy), as all children received concomitant interventions during their outpatient, day, or inpatient treatment at the treatment facility. However, parents, child psychologists, and child psychiatrists were requested not to make any changes in those interventions during data collection. Inclusion criteria for parents were the following: (a) no experience with PRT prior to this study, (b) access to a video camera and prepared to videotape him/herself and the child, and (c) ability to travel to the treatment facility during intervention. Informed consent was obtained from all parents and assent was obtained from children older than 12 years. The study was approved by the Ethics Committee of the Faculty of Social Sciences of the Radboud University, Nijmegen, the Netherlands (ECG2013-1304-100).

Child psychologists and child psychiatrists selected 31 parents and children for participation in group-based parent education in PRT. Of those, nine parents indicated that they did not want to participate in a parent education program. Six parents stated that they were willing to participate in parent education but gave no consent for participation in the study. The remaining 16 parents agreed to participate in group-based parent education and the study. Of those, three parents did not collect sufficient baseline data and were therefore excluded. Therefore, 13 parents and their children with ASD were included in the study.

Parents had a mean age of 43:8 years (*SD* = 5:7, range 36–52) and were primarily mothers (*n *= 9, 69%). Most parents were married or cohabiting (*n *= 11, 85%). Parent education ranged from high school (*n *= 7, 54%) to higher professional education or university (*n *= 6, 46%). Parents worked full-time (*n *= 4, 31%), part-time (*n* = 1, 8%), or were unemployed (*n* = 6, 46%). Two parents had another employment status (e.g., volunteer work). Children were aged between 8 and 14 years (*M *= 11:2, *SD* = 2:2) and were primarily boys (*n* = 10, 77%). All children had a clinical diagnosis of ASD according to the DSM-IV-TR or DSM-5 criteria. Mean score on the SRS was 82.2 (*SD* = 10.1, range 66–103) and average ADOS severity score was 6.8 (*SD* = 1.8, range 4–10). Seven children had one or two comorbid psychiatric disorders, including ADHD (*n* = 5), anxiety disorder (*n* = 1), posttraumatic stress disorder (*n *= 1), and ODD (*n* = 1). On average, children had a total IQ score of 98.0 (*SD* = 12.6, range 77–116), a verbal or reasoning IQ score of 99.7 (*SD* = 13.6, range 81–122), and a performance IQ score of 96.0 (*SD* = 13.3, range 75–113). Children received outpatient treatment (*n* = 1, 8%), day treatment (*n* = 5, 38%) or inpatient treatment (*n* = 7, 54%).

#### Design

A multiple baseline design across four groups of participants was used to investigate the effectiveness of group parent education in PRT on parent-created opportunities and child initiations (Kazdin 2011). Parents were randomly assigned to the first three groups and baseline started concurrently for these groups. For the fourth group, baseline started after the third intervention session of the third group. Parents were not randomly assigned to the fourth group. Participants remained in baseline for four, six, or eight weeks. Pre-tests and post-tests were conducted to explore the effectiveness of group parent education on parental stress and self-efficacy.

#### Procedures

##### Baseline

Baseline consisted of four to eight 10-min sessions. The purpose of baseline sessions was to assess the rate of parent-created opportunities for initiating prior to parent education in PRT and to assess the baseline level of child initiations. Baseline sessions were conducted at home and at the treatment facility if the child received inpatient treatment. During baseline, the parent did an age-appropriate everyday activity with the child that required interaction (e.g., playing a game, baking, or drawing). If the child initiated an interaction, the parent was instructed to respond as s/he usually did. Parents did not receive feedback on implementation of PRT techniques. They were asked to videotape baseline sessions using a video camera, tablet, or smartphone. Parents were instructed to record sessions (a) during which the parent, child, and activity were visible and audible on camera, (b) recorded in a one-to-one situation, and (c) lasting at least 10 min. If an activity lasted more than 10 min, parents were instructed to complete the activity. In addition, parents were asked to fill in two questionnaires during the last 2 weeks of baseline to measure parental stress and self-efficacy (see “Collateral Changes”).

##### Intervention

Group parent education was conducted by two child psychologists who worked at the specialized treatment center for children with ASD and were certified at PRT level III (i.e., demonstrated fidelity of PRT implementation at or greater than 80% with three children across three different activities). They had participated in staff training in PRT that was conducted by two PRT supervisors who were certified by the Koegel Autism Center. Each child psychologist had at least one year of experience in providing PRT. They were trained in providing parent education by the first author during a 3-h workshop.

The group parent education program consisted of eight 2-h group sessions and two 60-min individual sessions (Hardan et al. 2015; Minjarez et al. 2011). Sessions were conducted biweekly at the treatment facility. Groups were made up of both parents who did and did not consent to participate in this study. Groups consisted of seven to nine parents. From each group, two (group 3), three (group 2 and 4), or five parents (group 1) participated in this study. During group session 1–4, parents received instruction in PRT techniques (i.e., incorporating the child’s choice, gaining the child’s attention, providing clear opportunities, using contingent and natural reinforcement, reinforcing attempts, and interspersing maintenance and acquisitions tasks) via lectures, video-examples, worksheets, and role-plays. During the second group session, a (baseline) videotape was watched to assess the child’s current level of initiations and parents were taught to set goals for their child related to initiations. PRT had originally been developed to target functional initiations (e.g., protesting and requesting objects, help, or information), but in the present study parents also received instruction in techniques to elicit social initiations (e.g., requesting social information and commenting; Doggett et al. 2013). After each session, parents were instructed to practice PRT techniques during everyday activities with their child at home or at the treatment facility and videotape these sessions. During the first and second group session, parents did not receive feedback on their implementation of PRT in these videotapes, because the parents did not receive instruction in ‘incorporating the child’s choice’ and ‘gaining the child’s attention’ until the second group session. From the third group session, trainers provided feedback on parents’ implementation of PRT using the following video feedback protocol: (a) trainers and parents watched approximately 1 min of a videotape, (b) trainers provided the parent with positive performance-based feedback and praise for correct use of PRT techniques, (c) for incorrect or no use of the PRT techniques, trainers provided the parent with corrective performance-based feedback, asked the parent how he or she could have correctly used PRT techniques, and provided suggestions for correct use of PRT techniques if the parent did not give a correct answer, and (d) after watching about 5 min of a videotape, trainers concluded with a positive general comment on the parent’s performance (e.g., Robinson 2011). Individual sessions were conducted after the fourth and seventh group session. During these sessions, the child’s goals were evaluated and adjusted if necessary. Parents also received feedback on their implementation of PRT. During the eighth group session, the group parent education program was evaluated and parents were asked to fill in a questionnaire to assess the social validity of the group parent education program and PRT in general.

##### Post-intervention

We conducted three 10-min sessions immediately post parent education. Procedures were similar to those during baseline. During a post-intervention session, the parent did an age-appropriate everyday activity with the child that required interaction. Parents were instructed to implement PRT techniques but did not receive feedback on PRT implementation. The purpose of post-intervention sessions was to assess whether parents and children maintained their skills immediately after parent education. Four parents videotaped only one or two post-intervention sessions. Parents were also asked to fill out two questionnaires during the first 2 weeks of post-intervention to measure parental stress and self-efficacy (see “Collateral Changes”).

#### Dependent Measures

##### Parent-Created Opportunities

An event-recording system was used to measure parent-created opportunities for initiating (Cooper et al. 2013). A correct parent-created opportunity consisted of a sequence of correctly implemented PRT techniques (see Huskens et al. 2012; Verschuur et al. 2017). Three sequences were considered correct: (1) parent presenting a clear opportunity, child initiating, and parent reinforcing the child’s initiation contingently and naturally, (2) parent presenting a clear opportunity, parent prompting the child to initiate, child initiating, and parent reinforcing the child’s initiation contingently and naturally, and (3) parent presenting a clear opportunity, parent prompting the child to initiate at least once, child not initiating, and parent providing no reinforcement. Operational definitions of these behaviors are presented in Online Resource 1 (Carter and Hotchkis 2002; Huskens et al. 2012; Stahmer et al. 2011b; Verschuur et al. 2017). An example of a correct opportunity for a functional initiation would be the parent placing the mixer on a high shelf when making cupcakes with the child and immediately giving the child the mixer when he or she requested the mixer. An example of a correct opportunity to elicit a social initiation would be the parent stating *‘I’m going to cook something delicious tonight’* and answering that he or she will make lasagna when the child asked *‘What are we going to eat?’* An opportunity would also be correct if the parent controlled the bubble blower, prompted the child to ask for bubbles by saying ‘bubbles,’ and did not blow bubbles if the child did no reasonable attempt to say ‘bubbles’.

Ten minutes of each videotape were viewed and scored by observers naïve to the phase in which videotapes were recorded (i.e., baseline, intervention, or post-intervention). If videotapes lasted more than 10 min, 10 min in the middle were observed. The entire videotape was observed if a videotape lasted less than 10 min. Nine percent of videotapes lasted less than 10 min. Observers were instructed to record each sequence using numbers (e.g., 1 = statement, 2 = verbal model prompt, 3 = child initiation, and 4 = reinforcement) and to record the time point at which the parent presented a clear opportunity to determine interobserver agreement (see Interobserver agreement). Because 9% of the videotapes lasted less than 10 min, a rate of parent-created opportunities per minute was calculated by dividing the number of opportunities by the number of minutes.

##### Spontaneous Child Initiations

Spontaneous child initiations were measured using event-recording (Cooper et al. 2013). A spontaneous initiation was recorded if the child began or directed a social interaction to get a response from the parent without being prompted to initiate (Carter and Hotchkis 2002; Hauck et al. 1995; Wetherby 1986). The child began a social interaction if his or her verbal utterance occurred at least 5 s after parent’s last utterance or marked a change in conversation partner or activity. The child directed a social interaction if s/he changed or expanded the topic of conversation. Utterances that were part of an activity (e.g., ‘Does she have brown eyes?’ in the game Who is it?), play sounds (e.g., ‘vroom vroom’ when playing with cars), self-verbalizations, or echolalia were not recorded.

The function of each spontaneous initiation was recorded. Three functions were distinguished: functional, early social, and empathic social (Koegel et al. 2016b; Rieth et al. 2014). A functional initiation was recorded if the child’s utterance had the purpose of regulating the parent’s behavior, for example requesting objects (e.g., ‘Can I have the ball?’), requesting help (e.g., ‘Can you help me?’), or protesting (e.g., *‘*Will you stop it?’). Initiations were recorded as ‘early social’ if the child’s utterance had the purpose of initiating social conversation and called upon the parent’s involvement or interest, for example calling the parent’s attention (e.g., *‘*Mummy, look at my drawing!’) or giving information (e.g., ‘My classmate invited me to his birthday party next week’). An empathic social initiation was recorded if the child’s utterance had the purpose of initiating social conversation and indicated the child’s interest in the parent, for example seeking social information (e.g., ‘What movie did you see?’), or commenting (e.g., ‘That sounds great’).

Naïve observers viewed and scored 10 min of the videotapes. Observers were instructed to record the child’s initiation, the function of the initiation, and the time point at which the child initiated. A rate of spontaneous child initiations per minute was calculated for each function by dividing the number of spontaneous initiations by the number of minutes.

##### Collateral Changes

In order to explore whether group-based parent education in PRT led to collateral changes in parental stress and self-efficacy, two questionnaires were administered during baseline and post-intervention. The Dutch version of the Parenting Stress Questionnaire (PSQ) was used to measure parental stress (Vermulst et al. 2015). The PSQ is a 34-item questionnaire consisting of five subscales: (a) parent–child relationship problems, (b) parenting problems, (c) depressive mood, (d) parental role restriction, and (e) physical health problems. Items were rated on a four-point scale ranging from ‘not true’ to ‘very true’. Based on subscale scores a total score was calculated. Higher total scores indicate higher levels of parental stress. Evaluation of the psychometric qualities of the PSQ showed that the construct validity and internal consistency were good (Veerman et al. 2014).

The Dutch translation of the ‘Parental self-efficacy in the management of Asperger syndrome’ questionnaire was used to measure parental self-efficacy (Sofronoff and Farbotko 2002). This 15-item questionnaire describes 15 problem behaviors that are common in children with ASD (e.g., ‘When your child follows routines rigidly’). Parents indicated for each behavior whether their child displayed that behavior in the previous month and rated their confidence in managing the behavior on a six-point scale ranging from 0 (no confidence) to 5 (complete confidence). An average self-efficacy score was calculated by dividing the total rating of confidence by the number of problem behaviors displayed in the previous month.

##### Social Validity

During the last session of the parent education program, parents were asked to fill in a questionnaire to assess the social validity of group-based parent education and PRT in general. The questionnaire consisted of 29 statements (e.g., ‘The trainer’s feedback on my videotapes was useful’ and ‘The parent education program in PRT met my expectations’) that were rated on a five-point Likert scale ranging from 1 (strongly disagree) to 5 (strongly agree). These items measured parents’ attitude towards PRT, whether they judged the components of PRT parent education as informative and pleasant, and whether they considered the PRT parent education program to be effective. Finally, parents were asked to give an overall grade between 1 and 10 to rate the parent education program in PRT.

#### Interobserver Agreement

To determine interobserver agreement, 34% of the videotapes were coded by an independent and naïve second observer, approximately evenly distributed across parent–child dyads and study phases. Prior to coding videotapes, observers were trained to use the recording systems for parent-created opportunities and child initiations. Observer training included (a) discussion of definitions, examples, and non-examples using written guidelines and video examples, (b) practice of coding using 1-min segments, (c) discussion of any discrepancies and revision of written guidelines as necessary, and (d) independent coding of 10-min training videotapes (Ledford et al. 2018). Observer training continued until interobserver agreement was acceptable in two consecutive training videotapes (Cicchetti et al. 2006; Kennedy 2005). Ongoing training sessions were held every 3 weeks to minimize observer drift.

For parent-created opportunities, interobserver agreement was determined using mean count-per-interval (i.e., the average percentage of agreement across intervals; Cooper et al. 2013). Videotapes were divided into 10 one-minute intervals and for each interval a percentage of agreement between counts of both observers was calculated. Mean overall percentage of agreement (i.e., across videotapes) was 89% (*SD *= 13; range 40–100), indicating good interobserver agreement (Kennedy 2005), but interobserver agreement was less than 80% for 11% of the videotapes, indicating interobserver agreement was insufficient in several instances.

For spontaneous child initiations, interobserver agreement was determined using total count (Cooper et al. 2013). Total count interobserver agreement was calculated by dividing the lower count by the higher count multiplied by 100. Mean interobserver agreement using total count was 83% (*SD *= 15; range 33–100), indicating good interobserver agreement. However, 29% of the videotapes had interobserver agreement below 80%, indicating that interobserver agreement was insufficient in many videotapes. Based on spontaneous initiations that were recorded by both observers, interobserver agreement on the function of these initiations was assessed per category using prevalence-adjusted and bias-adjusted kappa (PABAK; Byrt et al. 1993). Mean PABAK was .77 (*SD* = .20), indicating good interobserver agreement (Cicchetti et al. 2006).

#### Treatment Integrity

An independent observer (i.e., a research assistant) collected data on treatment integrity based on audio recordings of the group sessions across the four groups using checklists. These checklists listed the parent education program components that were required to be implemented during a session (e.g., ‘The trainers provided instruction in the PRT technique ‘contingent and natural reinforcement’ using a PowerPoint presentation’). For each component, the independent observer recorded if the trainers did or did not correctly execute that component. Treatment integrity was calculated per session by dividing the number of correctly executed components by the total number of components multiplied by 100. Mean overall percentage of treatment integrity was 95% (*SD *= 10; range 50–100).

#### Data-Analysis

Analysis of data on parent-created opportunities and spontaneous child initiations involved visual and statistical analysis. Visual analysis was conducted to guide the statistical analysis (Parker and Vannest 2012) and consisted of systematic analysis of trend and level within and between subsequent phases for each participant, following the guidelines provided by Lane and Gast (2014). If a phase consisted of less than three data-points, no visual analysis was conducted (Kratochwill et al. 2010). Baseline trend was determined using the split-middle method of trend estimation. Median rates were compared to analyze changes in level between subsequent phases. Graphs that provide data on parent-created opportunities and spontaneous child initiations are presented in Online Resource 2.

Statistical analysis consisted of calculation of Tau_novlap_ or Tau-U (Parker et al. 2011). Both are effect sizes for single-case research that examine the proportion of non-overlap of data between two subsequent phases, but in addition, Tau-U also controls for undesirable positive baseline trends. If visual analysis indicated a strong positive baseline trend, Tau-U was calculated. Tau and the corresponding *p* value were calculated for the baseline/intervention-contrast and intervention/post-intervention-contrast for each participant using Single Case Research (SCR), a web-based calculator (Vannest et al. 2016). Because post-intervention consisted of less than three data points for four participants, no Tau was calculated for the intervention/post-intervention contrast for these participants (Kratochwill et al. 2010). Overall effect sizes (i.e., across parents or children) and confidence intervals were also calculated for both phase contrasts using SCR. Analyses were two-tailed and *p* value was set at .05. Using the guidelines of Vannest and Ninci (2015), overall effect sizes were interpreted as small (≤ .20), moderate (.21–.60), large (.61–.80), or very large (≥ .81).

Data on parental stress were analyzed for each parent using the reliability of change index (RCI; Jacobson and Truax 1991). The RCI indicates whether changes in scores between baseline and post-intervention were statistically significantly greater than differences in scores that could have occurred due to random measurement error alone. The RCI was calculated using the following formula:

$$RCI = \frac{{X_{2} - X_{1} }}{{\sqrt {2(S_{1} \sqrt {1 - r_{xx} )} } }}$$

where *X*_*1*_ en *X*_*2*_ represent the baseline and post-intervention PSQ total scores, *S*_*1*_ the standard deviation of the reference sample, and *r*_*xx*_ the test–retest reliability of the PSQ, as reported in the manual (Vermulst et al. 2015). Analyses were two-tailed and *p*-value was set at .05. Consequently, an RCI < − 1.96 indicated reliable negative change and a significant decrease in parental stress. An RCI > 1.96 indicated reliable positive change and a significant increase in parental stress (Jacobson and Truax 1991). Data on parental self-efficacy were not analyzed using the RCI, because no test–retest reliability was available for the questionnaire. In addition to the RCI, data on both parental stress and self-efficacy were analyzed using Wilcoxon signed-rank tests to determine whether overall (i.e., across parents) statistically significant changes occurred as a result of parent education. In order to determine the magnitude of the changes, effect sizes were also calculated (Cohen 1992). Following Cohen’s guidelines, *r* was interpreted as small (.10), medium (.20), or large (.50).

Spearman rank correlation coefficients were calculated to assess the association between changes in parent-created opportunities and other dependent measures (i.e., functional, early social, and empathic social initiations, parental stress, and self-efficacy) between baseline and intervention or post-intervention.

### Results

#### Parent-Created Opportunities

Median rates of parent-created opportunities during baseline, intervention, and post-interventions and values of Tau are provided in Online Resource 3. During baseline, the median rate of parent-created opportunities ranged from 0.00 to 0.10, indicating that parents hardly created opportunities for initiating prior to parent education in PRT. During intervention, the rate of parent-created opportunities increased significantly for six (out of 13) parents. The combined Tau across parents was .52 (90% CI .36–.67, *p* < .001), indicating a moderate significant effect of group parent education in PRT on parent-created opportunities. During post-intervention, no significant changes in the rate of parent created opportunities occurred, suggesting that parents maintained their skills after parent education.

#### Spontaneous Child Initiations

During baseline, the median rate of functional initiations ranged from 0.30 to 1.50 (see Online Resource 3), indicating that most children already produced functional initiations prior to parent education in PRT. During intervention, the rate of functional initiations increased significantly for three (out of 13) children. The combined Tau across children was .37 (90% CI .23–.52, *p* < .001), indicating a moderate significant effect of group-based parent education in PRT on functional initiations. During post-intervention, the rate of functional initiations did not change significantly, suggesting that children maintained their intervention level of functional initiations.

There were no significant increases in the rate of early social initiations during intervention or post-intervention. However, early social initiations decreased significantly for one child (out of 13 children) during intervention.

For empathic social initiations, the median baseline rate ranged from 0.00 to 0.75 (see Online Resource 3), but most children hardly produced empathic social initiations during baseline. During intervention, the rate of empathic social initiations significantly increased for one (out of 13) children. The combined Tau across children was .26 (90% CI .12–.41, *p* = .003), indicating a moderate significant effect of group parent education in PRT on empathic social initiations. From intervention to post-intervention, the rate of empathic social initiations increased significantly for one child (out of nine children) and overall, most children maintained their intervention level of empathic social initiations during post-intervention.

**Fig. 1:**
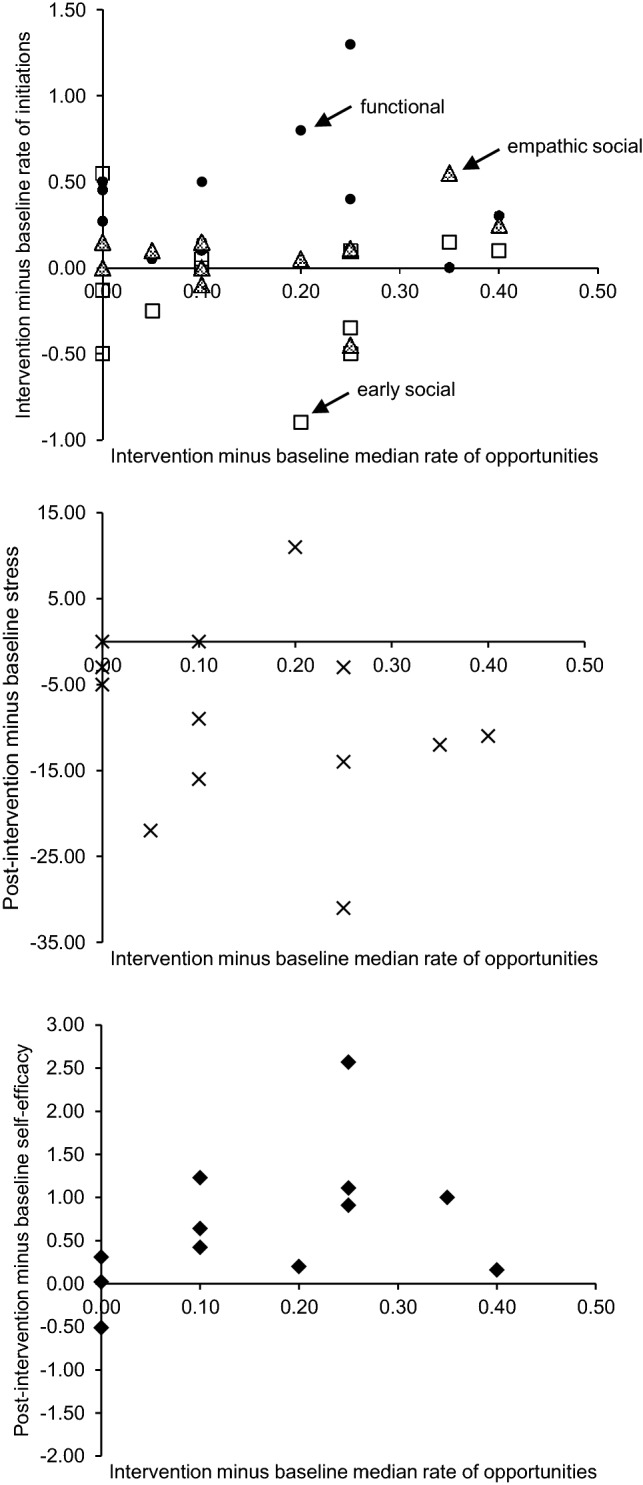
Relationship between changes from baseline to intervention/post-intervention in parent-created opportunities and functional initiations (circles), early social initiations (squares), and empathic social initiations (triangles; top), parental stress (crosses; middle), and parental self-efficacy (diamonds; bottom) for group parent education

The top scatterplot in Fig. 1 presents the relationship between changes in median rates of parent-created opportunities from baseline to intervention and changes in median rates of functional, early social, and empathic social initiations. Changes in parent-created opportunities were not significantly associated with changes in functional initiations (*r*_*s*_ = .06, *p* = .86), early social initiations (*r*_*s*_ = − .03, *p* = .91), or empathic social initiations (*r*_*s*_ = .30, *p* = .32).

#### Collateral Changes

Parental stress reduced reliably and significantly in eight parents, did not change significantly in four parents, and increased significantly in one parent (out of 13 parents). Across parents, there was a medium to large significant decrease in stress from baseline (*Mdn* = 75) to post-intervention (*Mdn* = 61), *z* = − 2.45, *p* = 0.01, *r *= − 0.48. Changes in parental stress were not significantly related to changes in parent-created opportunities (*r*_*s*_= .28*, p* = .36; see Fig. 1). There was a large significant increase in self-efficacy across parents between baseline *(Mdn* = 3.47) and post-intervention (*Mdn *= 4.00), *z* = − 2.59, *p* = 0.01, *r *= − 0.53. Changes in self-efficacy were not significantly associated with changes in parent-created opportunities (*r*_*s*_= − .44*, p* = .15; see Fig. 1).

#### Social Validity

Twelve (out of 13) parents completed the social validity questionnaire. Parents rated the group parent education program in PRT as highly informative (*M *= 4.39) and pleasant (*M *= 3.87). Components that were rated most informative were practicing at home (*M* = 4.73) and video-feedback (*M* = 4.58). Role-plays were rated least informative (*M* = 3.58). After group parent education in PRT, parents had a positive attitude towards PRT (*M* = 4.42) and indicated that group-based parent education benefited their child’s development (*M* = 4.25) and their interaction with their child (*M* = 4.25). Overall, parents rated the group parent education program in PRT with an 8.00.

## Study 2: Individual Parent Education in PRT

### Method

#### Participants and Setting

Study 2 was conducted in two community-based treatment facilities for children with ASD in the Netherlands. The first treatment facility was the same specialized treatment center for children with ASD as in Study 1 (i.e., Dr. Leo Kannerhuis). The second treatment facility was Youké, which is a center for youth care that also provided day treatment to children with ASD. Child psychologists and child psychiatrists in both treatment facilities referred potential participants to the study. Inclusion and exclusion criteria for parents and their children were identical to those in Study 1. The study was approved by the Ethics Committee of the Faculty of Social Sciences of the Radboud University, Nijmegen, the Netherlands (ECG2013-1304-100).

Child psychologists and child psychiatrists selected 20 parents and children for participation in individual parent education in PRT. Of those, 16 parents agreed to participate in individual parent education and the study. One of these parents was excluded because the child’s IQ was too low. Two parents were excluded because they did not collect sufficient baseline data. Therefore, 13 parents and their children with ASD were included in the study to investigate the effectiveness of individual parent education in PRT. Six children received treatment at the first treatment facility and seven children were treated at the second treatment facility.

Parents had a mean age of 40:6 years (*SD* = 7:8, range 30–55) and were primarily mothers (*n *= 9, 69%). Most parents were married or cohabiting (*n *= 10, 77%). Parent education ranged from high school (*n *= 6, 46%) to higher professional education or university (*n *= 7, 54%). Parents worked full-time (*n *= 3, 23%), part-time (*n* = 8, 62%), or were unemployed (*n* = 2, 15%). Children had a mean age of 6:11 years (*SD* = 3:2; range 3–11) and were primarily boys (*n* = 11, 85%). All children had a clinical diagnosis of ASD according to the DSM-IV-TR or DSM-5 criteria. Mean score on the SRS was 84.0 (*SD* = 8.5, range 76–109) and average ADOS severity score was 6.1 (*SD* = 1.9, range 3–9). Three children had one or two comorbid psychiatric disorders, including ADHD (*n* = 1), anxiety disorder (*n* = 1), ODD (*n* = 1), and unspecified conduct disorder (*n* = 1). On average, children had a total IQ score of 95.4 (*SD* = 14.0, range 72–119), a verbal or reasoning IQ score of 98.3 (*SD* = 9.6, range 87–115), and a performance IQ score of 90.2 (*SD* = 17.6, range 59–122). Children received outpatient treatment (*n* = 1, 8%), day treatment (*n* = 9, 69%) or inpatient treatment (*n* = 3, 23%).

#### Design

Two concurrent multiple baseline designs across participants (i.e., one for each treatment facility) were used to investigate the effectiveness of individual parent education (Kazdin 2011). Participants were randomly assigned to a baseline length and remained in baseline for four, six, or eight weeks. Pre-tests and post-tests were conducted to explore the effectiveness of individual parent education on parental stress and self-efficacy.

#### Procedures

##### Baseline

Baseline consisted of four to eight 10-min sessions. Procedures for baseline sessions were identical to those in Study 1.

##### Intervention

Individual parent education was conducted by a child psychologist, social worker, or direct-care staff member who worked at the first or second community-based treatment facility. Each trainer was certified at PRT level III and had at least 1 year of experience in providing PRT. Trainers were trained in providing individual parent education by the first author during a 3-h workshop.

Individual parent education consisted of four 90-min sessions and six 60-min sessions. Sessions were conducted weekly at one of the treatment facilities or at the parents’ home. Sessions were attended by one parent or both parents. Parents received instruction in PRT techniques via lectures, video-examples, worksheets, and role-plays during session 1–4. Children did not attend these sessions, since these sessions were designed to provide parents with instruction in PRT techniques. During the second session, a (baseline) videotape was watched to assess the child’s current level of initiations and parents were taught to set goals for their child related to initiations. After each session, parents were instructed to practice PRT techniques during everyday activities with their child and to videotape these sessions. During the first and second session, parents did not receive feedback on their implementation of PRT in these videotapes, because parents did not receive instruction in ‘incorporating the child’s choice’ and ‘gaining the child’s attention’ until the second session. As of the third session, parents received feedback on their implementation of PRT techniques using the same video feedback protocol as was used in Study 1. Sessions 5–10 were also attended by the child. During the first 10 min of these sessions, parents practiced PRT techniques with their child during an everyday activity, which was videotaped by the trainer. These PRT sessions were conducted in case the parent had not been able to videotape a PRT session at home. During the next 30 min, parents received feedback on their implementation of PRT in the videotape recorded by the trainer and/or the videotape recorded at home. For the remainder of the session, parents practiced PRT techniques with their child during an everyday activity while receiving feedback from the trainer (i.e., guided practice) using the following protocol: (a) if the parent implemented PRT correctly, the trainer provided positive performance-based feedback and praise every minute, (b) if the parent implemented PRT incorrectly or did not implement PRT, the trainer provided corrective performance-based feedback and modeled correct use of PRT, and (c) after 20 min, the trainer concluded with a positive general comment on the parent’s performance (e.g. Randolph et al. 2011). During the fifth and ninth session, the child’s goals were evaluated and adjusted. During the tenth session, the individual parent education program in PRT was evaluated and parents were asked to complete a questionnaire to assess the social validity of individual parent education and PRT in general.

##### Post-intervention

Post-intervention consisted of three 10-min sessions that were conducted immediately post parent education. Procedures for post-intervention sessions were identical to those in Study 1. One parent videotaped only two post-intervention sessions and another parent was not able to record any post-intervention sessions.

#### Dependent Measures

Dependent measures were identical to those in Study 1.

#### Interobserver Agreement

To determine interobserver agreement, 36% of the videotapes were coded by an independent and naïve second observer, approximately evenly distributed across parent–child dyads and study phases. Prior to coding videotapes, observers were trained to use the recording systems for parent-created opportunities and child initiations. This observer training was the same as in Study 1.

For parent-created opportunities, interobserver agreement was determined using mean count-per-interval (Cooper et al. 2013). Mean overall percentage of agreement was 86% (*SD *= 13; range 40–100), indicating good interobserver agreement (Kennedy 2005), but interobserver agreement was less than 80% for 28% of the videotapes, indicating that interobserver agreement was insufficient in many videotapes.

For spontaneous child initiations, interobserver agreement was determined using total count (Cooper et al. 2013). Mean interobserver agreement using total count was 79% (*SD *= 18; range 17–100), indicating acceptable interobserver agreement. However, 40% percent of the videotapes had interobserver agreement below 80%, indicating that interobserver agreement was insufficient in many instances. Based on initiations that were recorded by both observers, interobserver agreement on the function of these initiations was assessed per category using PABAK (Byrt et al.^1993^). Mean PABAK was .77 (*SD* = .20), indicating good interobserver agreement (Cicchetti et al. 2006).

#### Treatment Integrity

Each trainer collected data on treatment integrity after each session using checklists. These checklists listed the individual parent education program components that were required to be implemented during a session (e.g., ‘During video-feedback, the trainer gave positive performance-based feedback and praised the parent every minute, if he or she created one or more correct opportunities’). For each component, the trainer recorded if he or she did or did not correctly execute that component. Mean overall percentage of treatment integrity was 95% (*SD *= 8; range 63–100). For 21% of the sessions an independent observer (i.e., research assistant) collected data on treatment integrity based on audio recordings of the sessions to assess interobserver agreement in treatment integrity using PABAK. Mean value of PABAK was .82 (*SD* = .23), indicating excellent interobserver agreement on treatment integrity (Cicchetti et al. 2006).

#### Data-Analysis

Analyses were identical to those in Study 1.

### Results

#### Parent-Created Opportunities

The baseline median rate of parent-created opportunities ranged from 0.00 to 0.35 (see Online Resource 3), indicating that most parents did not create opportunities for initiating prior to individual parent education in PRT. During intervention, the rate of parent-created opportunities increased significantly for 11 (out of 13) parents. The combined Tau across parents was 0.80 (90% CI 0.66–0.94, *p* < .001), indicating a large effect of individual parent education in PRT on parent-created opportunities. During post-intervention, there was a significant increase in the rate of parent-created opportunities for one (out of 11) parents, but across parents the rate of parent-created opportunities hardly changed (Tau_combined_ = 0.04, 90% CI − 0.15–0.24, *p* = .71). This suggests that, overall, parents maintained their skills after parent education.

#### Spontaneous Child Initiations

During baseline, the median rate of functional initiations ranged from 0.00 to 1.30 (see Online Resource 3), indicating that most children already produced functional initiations prior to parent education. During intervention, the rate of functional initiations increased significantly for 10 (out of 13) children. The combined Tau across children was .65 (90% CI .52–.79, *p* < .001), indicating a large significant effect of individual parent education on functional initiations. During post-intervention, there were no significant changes in the rate of functional initiations (see Online Resource 3), suggesting that, overall, children maintained their intervention level of functional initiations.

There were no significant increases in the rate of early social initiations during intervention. However, early social initiations decreased significantly for one child (out of 13 children). During post-intervention, there was an increase in the rate of early social initiations for two (out of 11) children.

For empathic social initiations, the median baseline rate ranged from 0.00 to 0.15 (see Online Resource 3), indicating that children hardly produced empathic social initiations during baseline. During intervention, the rate of empathic social initiations significantly increased for two (out of 13) children. The combined Tau across children was .13 (90% CI .00–.27, *p* = .10), indicating a small non-significant effect of individual parent education on empathic social initiations. From intervention to post-intervention, there were no significant changes in the rate of empathic social initiations, suggesting that, overall, children maintained their intervention level of empathic social initiations during post-intervention.

**Fig. 2:**
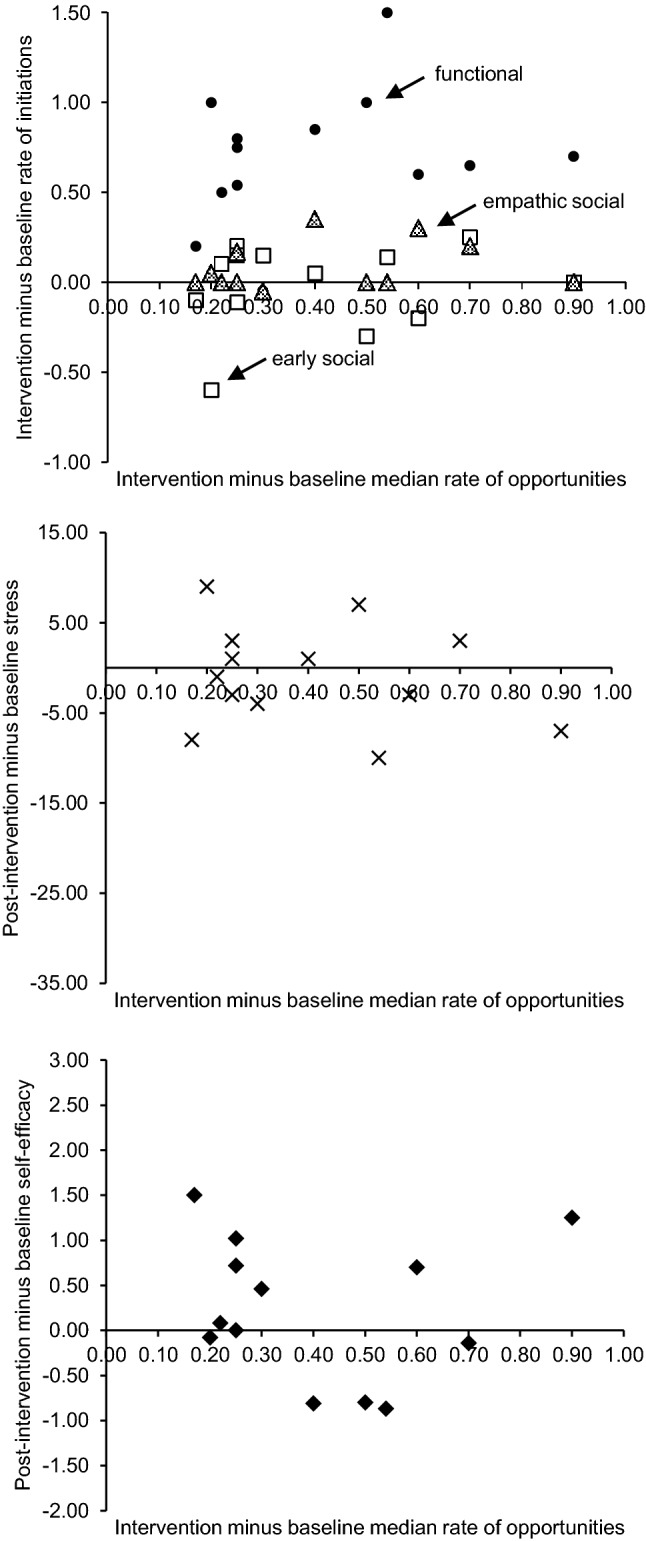
Relationship between changes from baseline to intervention/post-intervention in parent-created opportunities and functional initiations (circles), early social initiations (squares), and empathic social initiations (triangles; top), parental stress (crosses; middle), and parental self-efficacy (diamonds; bottom) for individual parent education

The top scatterplot in Fig. 2 presents the relationship between changes in median rates of parent-created opportunities from baseline to intervention and functional, early social, and empathic social initiations for individual parent education. There was no significant association between changes in parent-created opportunities and changes in functional initiations (*r*_*s*_ = .27 *p* = .38), early social initiations (*r*_*s*_ = .16, *p* = .61), or empathic social initiations (*r*_*s*_ = .23, *p* = .44).

#### Collateral Changes

Parental stress reduced reliably and significantly in three parents, did not change significantly in eight parents, and increased significantly in two parents (out of 13 parents). Across parents, there was no significant change in stress between baseline (*Mdn *= 61) and post-intervention (*Mdn *= 62), *z* = − 0.63, *p* = 0.53, *r *= − 0.12. Changes in parental stress were not significantly related to changes in parent-created opportunities (*r*_*s*_= .14*, p* = .64; see Fig. 2). There was no significant increase in self-efficacy across parents from baseline (*Mdn *= 3.73) to post-intervention (*Mdn *= 3.78), *z* = − 0.82, *p* = 0.41, *r *= − 0.16. Changes in self-efficacy were not significantly associated with changes in parent-created opportunities (*r*_*s*_= .24*, p* = .40; see Fig. 2).

#### Social Validity

All parents completed the social validity questionnaire after parent education. Individual parent education was rated as highly informative (*M* = 4.42) and pleasant (*M* = 4.23). With regard to the components of the individual parent education program, video-feedback and guided practice were rated most informative, with mean scores of 4.69 and 4.54, respectively. Role-plays were rated least informative, but nonetheless highly informative (*M* = 4.23). Parents’ attitude towards PRT was positive post parent education (*M* = 4.23) and parents indicated that individual parent education in PRT benefited their child’s development (*M* = 4.08) and their interaction with their child (*M* = 4.08). Overall, parents rated the individual parent education program in PRT with an 8.08.

## Discussion

In two single-case design studies, parents of children with ASD were taught to create opportunities for initiations through group or individual parent education in PRT. Collateral changes in parental stress and self-efficacy as a result of group or individual parent education were also explored. The results from the first study indicate that group-based parent education in PRT had a moderate significant effect on parent-created opportunities, functional initiations, and empathic initiations. Furthermore, parental stress significantly decreased and self-efficacy significantly increased. Finally, parents were highly satisfied with the group parent education program. The results from the second study show that parents were also highly satisfied with individual parent education in PRT. Moreover, individual parent education resulted in large significant increases in parent-created opportunities and functional initiations, but changes in parental stress and self-efficacy were not significant. In both studies, changes in parent-created opportunities were not significantly associated with changes in any other dependent measure.

The results from the first study partially support the notion that parents can effectively be taught PRT in a group in a community-based treatment facility. Less than half of the parents created significantly more opportunities as a result of group-based parent education and functional and empathic social initiations increased significantly in only a few children, despite moderate effects across parents and children. One factor that could explain why our group parent education program in PRT seems less effective than those in the studies of Hardan et al. (2015) and Minjarez et al. (2011) is the age of the included children. Children of parents in our group parent education program were older (i.e., school-aged). PRT might be more difficult to implement in school-aged children, because these children play or work more independently compared to preschoolers, which might make it more difficult to create opportunities for functional initiations (Suhrheinrich et al. 2016). Also, more than half of the children in our group parent education program had at least one comorbid psychiatric diagnosis, whereas children with comorbid psychopathology were excluded from participation in the study of Hardan et al. (2015). Comorbid psychopathology may negatively affect the effectiveness of an intervention (e.g., Antshel et al. 2011). Furthermore, comorbid psychopathology is often the primary reason for referral to treatment services and thus, parents may prioritize intervention targets related to these co-occurring psychiatric problems over initiations (e.g., Brookman-Frazee et al. 2012a). Another factor that might explain the mixed results of our study on group parent education is the amount of practice and individual feedback. On average, parents who participated in group-based parent education videotaped only seven sessions during parent education, although they were instructed to videotape one PRT session after each session. Thus, parents in our group parent education program practiced less than instructed, suggesting low treatment adherence. As a result, they received less feedback. Both practice and individual feedback are critical to enhance parents’ skills (e.g., Parsons et al. 2012). It is possible that parents in group-based parent education felt little pressure to practice because they could ‘hide behind’ the group (Wymbs et al. 2017). For future research it is important to determine the optimum amount and type of practice and feedback to teach parents to create opportunities for initiating and to identify strategies to increase treatment adherence in group parent education programs.

Findings from our second study are consistent with previous studies on individual parent education in PRT that have shown increases in parents’ ability to implement PRT and improvements in child functional verbal communication, including initiations (e.g., Bradshaw et al. 2017; Coolican et al. 2010; Koegel et al. 2002; Randolph et al. 2011; Symon 2005). The current study provides additional evidence for the effectiveness of individualized parent education programs in PRT by demonstrating that individual parent education can be effectively implemented by trainers in community-based treatment facilities.

In both studies changes in parent-created opportunities were not significantly related to collateral changes in parental stress and self-efficacy. Rather, particularly in our study on parent education using a group model, improvements in parental stress and self-efficacy seemed to occur irrespective of increases in parent-created opportunities. This suggests that it may be important to provide parents with opportunities to meet other parents of children with ASD who have similar experiences, prior or in addition to teaching skills to these parents to improve their child’s skills. As such, these results support the hypothesis that participating in parent education in a group context may decrease parental stress and increase their self-efficacy, because a parent education group would increase social support (e.g., Frantz et al. 2018).

Our studies on group and individual parent education in PRT are unique in distinguishing between different subtypes of child initiations based on their communicative function compared to other studies evaluating the effectiveness of PRT on child initiations. Until now, studies on PRT targeted only one type of initiations (e.g., Koegel et al. 2010) or initiations in general without distinguishing between communicative functions (e.g., Hardan et al. 2015; Minjarez et al. 2011). A distinction between initiations based on their communicative function is important as social initiations have more potential to improve children’s social success than functional initiations (e.g., Koegel 2000). PRT particularly provides strategies to elicit functional child initiations (e.g., shared control, waiting, and interrupting a routine), although leading statements can be used to create opportunities for empathic social initiations (e.g., Doggett et al. 2013). Therefore, it is not surprising that we particularly found significant increases in functional and to a lesser degree in empathic social initiations as a result of parent education in PRT. Our baseline data also suggest that children with ASD may show deficits in one subtype of initiations, but not in another subtype. These data support the notion that PRT needs to be individualized based on child characteristics and that parents need to be taught to create opportunities to target a certain subtype of initiations (Rieth et al. 2014). Further research is necessary to validate our distinction in subtypes of initiations.

There was a great deal of variability in responding between parents and children in both studies. Parent characteristics may influence parents’ intervention outcomes, including parent fidelity of intervention implementation (e.g., Randolph et al. 2011). For example, parent education may be less effective in economically disadvantaged families, although these parents appeared to benefit significantly more from individual than group parent education models (Lundahl et al. 2006). Parent’s cultural background, educational level, marital status, parental stress, psychopathology, and gender are also likely to be related to parent fidelity of intervention implementation (e.g., Osborne et al. 2008; Reyno and McGrath 2006; Stahmer et al. 2011a; Strauss et al. 2012). Further research is necessary to examine how these factors affect the effectiveness of parent education in PRT. This will enable clinicians to tailor parent education in PRT to each parent’s needs and to optimize individual outcomes.

Variability in responding between children may be a result of variation in parents’ implementation of PRT as fidelity of implementation is associated with intervention outcomes (e.g., Allen and Warzak 2000; Strauss et al. 2012). Furthermore, child characteristics might account for this variability. Research has indicated that higher pre-intervention cognitive and expressive language skills, more positive affect, more appropriate toy contact, and decreased social avoidance and stereotyped or repetitive vocalizations predict positive outcome of PRT in preschool children with ASD (Fossum et al. 2018; Schreibman et al. 2009; Sherer and Schreibman 2005). Future research should investigate whether these and other child characteristics, such as comorbid psychopathology and challenging behavior, are related to outcomes of PRT for school-aged children with ASD.

There are several limitations to both studies. First, due to limited availability of certified PRT trainers, the studies on group and individual parent education could not be conducted concurrently. As a result, parents and children were not randomly assigned to the study on group or individual parent education or matched. We conducted two separate single-case design studies and thus, differences in the effectiveness of both parent education models could not be evaluated. This topic should be addressed in future research. Second, children included in both studies were also receiving other interventions at the treatment facilities (e.g., milieu therapy, speech-language therapy, art therapy, family therapy, pharmacological interventions, or physiotherapy). For ethical reasons, it was necessary to continue these interventions and thus the possibility of multiple intervention interference cannot be ruled out. Third, although interobserver agreement was acceptable or good on average, there were several instances where interobserver agreement was below acceptable levels, which impacts the accuracy of our data. Interobserver agreement for parent-created opportunities may have been low in several instances, because a parent-created opportunity was defined as a sequence of multiple behaviors (Cooper et al. 2013). Because we measured spontaneous initiations in children with very different levels of verbal communication (ranging from a few words to verbally fluent) and measured different types of initiations (i.e., functional, early social, and empathic social), our definition of an initiation was relatively ‘broad’, which may have resulted in low levels of interobserver agreement in many videotapes (Cooper et al. 2013). In addition, interobserver agreement may have been low, because the audio quality of several videotapes was poor due to background noises or bad acoustics. Fourth, to practice the PRT techniques parents were allowed to choose any age-appropriate everyday activity that required interaction. This enabled parents to select child-preferred activities and to follow their child’s motivation, as is expected during PRT (Koegel et al. 2016a), but also resulted in variation in activities, which could have affected our results. Finally, collateral effects were measured only before and after the intervention using questionnaires completed by parents. Repeated measures using a combination of direct or objective assessment methods (e.g., observation or physiological measures) and indirect or subjective measures (e.g., questionnaires) would have been more suitable to measure changes in parental stress and self-efficacy (Cooper et al. 2013).

Despite the above stated limitations, this study provides support for the use of individual parent education in PRT in community-based treatment facilities to teach parents to create opportunities for initiations. Our findings also suggest that delivering parent education in a group format is moderately effective for this purpose. Also, providing parents with opportunities to meet with other parents in similar circumstances may result in reductions in parental stress and increases in self-efficacy. As current demand for treatment services for children with ASD exceeds the availability of such services and effective and efficient interventions are essential, clinicians in community-based treatment facilities may choose to combine individual parent education in PRT with group sessions. Further research is warranted to identify parent and child characteristics that affect effectiveness of parent education in PRT in order to be able to tailor interventions to meet each individual’s needs and to optimize individual outcomes.

## Electronic supplementary material

Below is the link to the electronic supplementary material.

Supplementary material 1 (PDF 753 kb)
