# Supplementary material for: Effectiveness of Parent Education in Pivotal Response Treatment on Pivotal and Collateral Responses
Source: J Autism Dev Disord. 2019 May 24;49(9):3477–93. doi: 10.1007/s10803-019-04061-6 (PMC6667419; doi:10.1007/s10803-019-04061-6)
Supplement: Supplementary file 1 — Supplementary material 1 (PDF 753 kb) [file 10803_2019_4061_MOESM1_ESM.pdf]

SUPPLEMENTARY MATERIALS

Article: Effectiveness of parent education in Pivotal Response Treatment on pivotal and collateral responses

Journal: Journal of Autism and Developmental Disorders

Authors: R. (Rianne) Verschuur, B. (Bibi) Huskens, and R. (Robert) Didden

Correspondence: Rianne Verschuur, Behavioural Science Institute, Radboud University, P.O. Box 9104, 6500 HE Nijmegen, The Netherlands. E-mail: r.verschuur@pwo.ru.nl.

Online Resource 1. Definitions of behavioral categories for parent-created opportunities

Behavioral category	Operational definition
1. Parent presenting a clear opportunity	The parent presented a clear opportunity (i.e., the first presentation of a signal for the child to initiate) by:<br>(a) Shared control: the parent had control over an object the child desired or needed during the activity.<br>(b) In sight, out of reach: the object the child desired or needed during the activity was visible, but out of the child's reach; the object was neither in the parent's possession.<br>(c) Out of sight, out of reach: the object the child desired or needed during the activity was invisible and out of the child's reach; the object was neither in the parent's possession.<br>(d) Waiting: the parent did nothing and waited for three seconds, when the routine of the activity expected the parent to act or when the child needed help to carry out an action<br>(e) Interrupting a routine: the parent did something that did not fit in the routine of the activity, for example throwing the dice while it was the child's turn.<br>(f) Carrier phrase: the parent sang a familiar song or said a familiar rhyme/saying, but omitted the last word, for example *'Ready, set ...'* and then remained silent.<br>(g) Making a leading statement: the parent made a statement or comment without giving details to the child, for example *'I have done something fun yesterday'*, and then remained silent.
2. Child initiating	The child began or directed a social interaction to get a response from the parent, spontaneously or prompted, for example *'Ball?'* or *'Can you help me?'* or *'What movie did you see?'*
3. Parent prompting the child to initiate	If the child did not initiate within 5 seconds after the parent presented a clear opportunity, the parent offered help by prompting. Three types of prompts were recorded:<br>(a) Time delay prompt: the parent was silent for three seconds, while giving the child a questioning look and/or making a sign to stimulate the child to initiate.<br>(b) Open-ended question prompt: the parent asked an open question to stimulate the child to initiate, for example *'What could you ask me now?'*<br>(c) Verbal model prompt: the parent modeled verbal utterance that the child could use to initiate.<br>The parent continued prompting until the child initiated, until the parent gave three prompts (children who were not verbally fluent) or until a verbal model prompt was given (children who were verbally fluent).
4. Parent reinforcing the child's initiation contingently and naturally	The parent reinforced the child's initiations naturally and contingently by responding to this initiation within two seconds. Contingent and natural reinforcement was recorded if (a) the response was the parent's first behavior after the child's initiation and (b) the response was a natural consequence of the child's initiation. Natural reinforcers could be both tangibles (e.g., the child receives an object) or verbal responses (e.g., the child gains social information). Contingent and natural reinforcement was also recorded if the parent withheld reinforcement if the child did not initiate after being prompted at least once.

Online Resource 2. Graphs providing data on parent-created opportunities and spontaneous child initiations.

Figure 1. Rate of parent-created opportunities (group parent education)

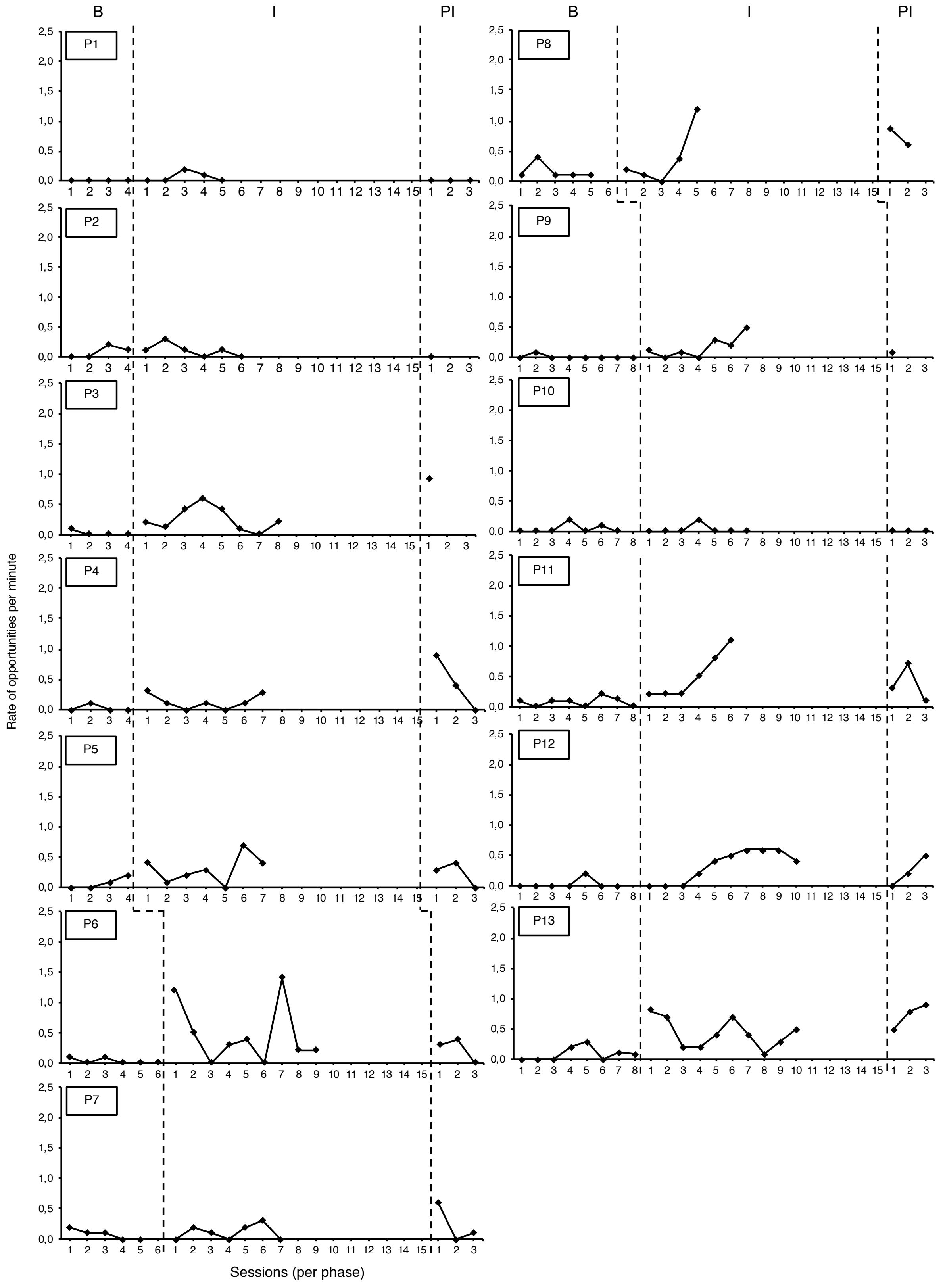

Figure 2. Rate of functional, early social, and empathic social initiations (group parent education)

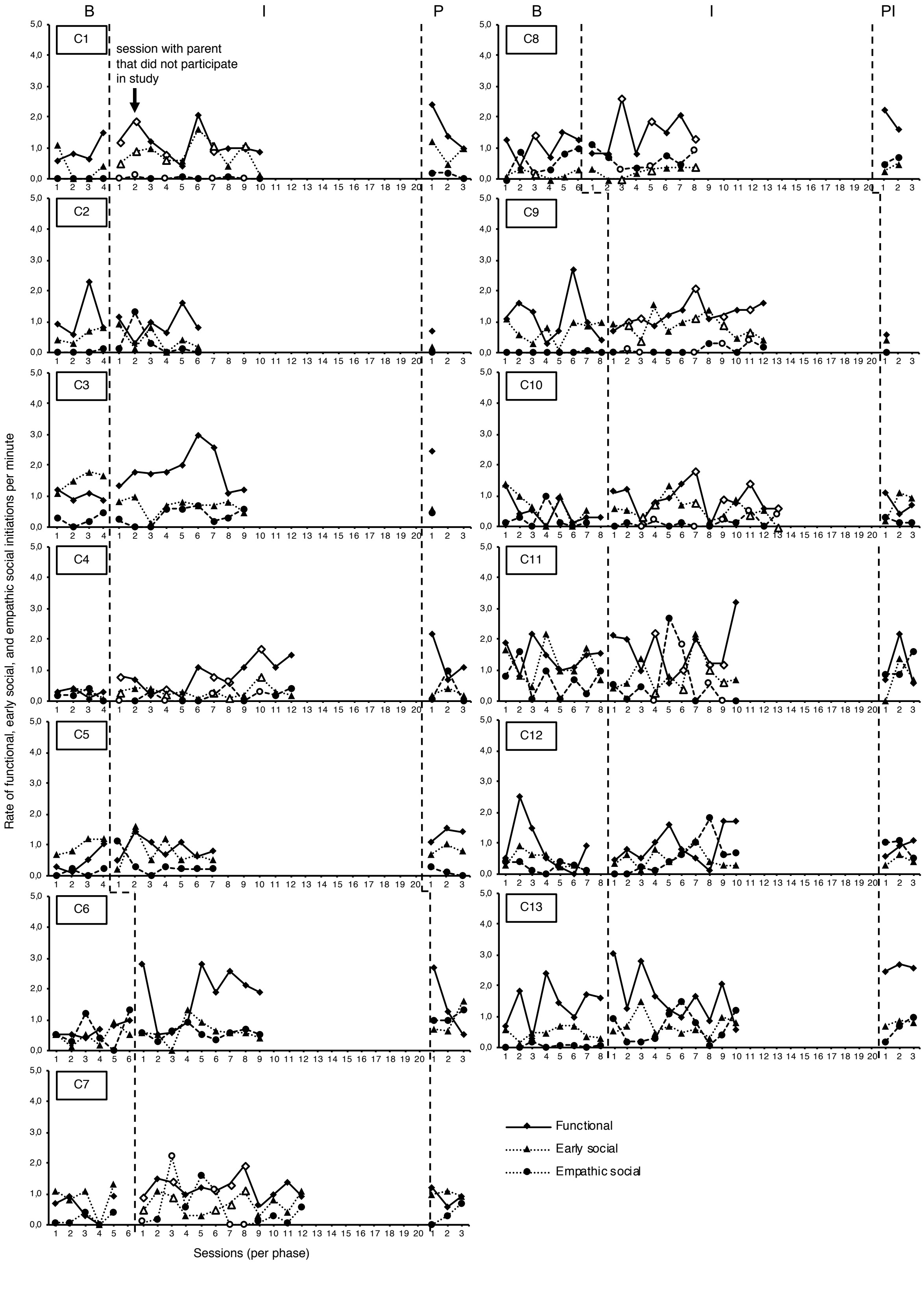

Figure 3. Rate of parent-created opportunities (individual parent education, treatment facility 1)

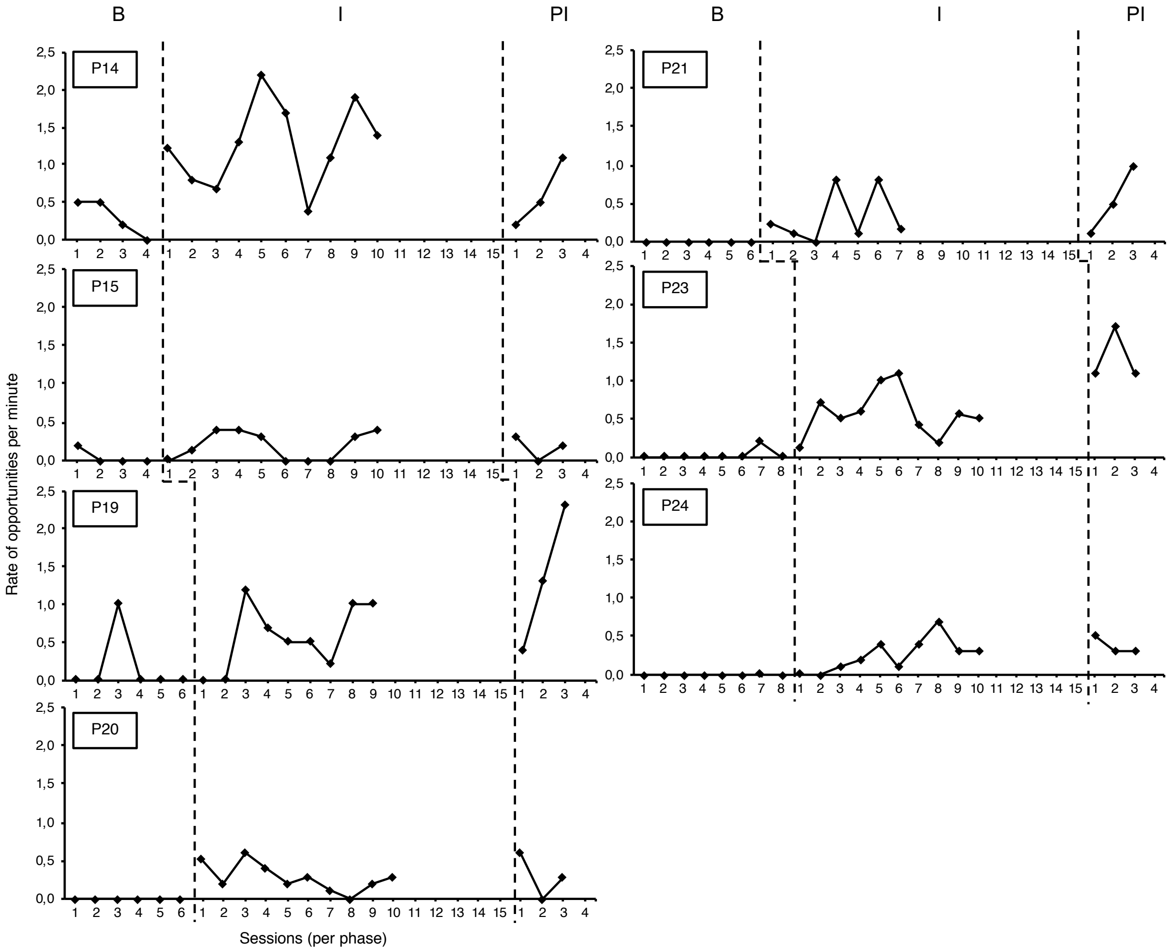

Figure 4. Rate of parent-created opportunities (individual parent education, treatment facility 2)

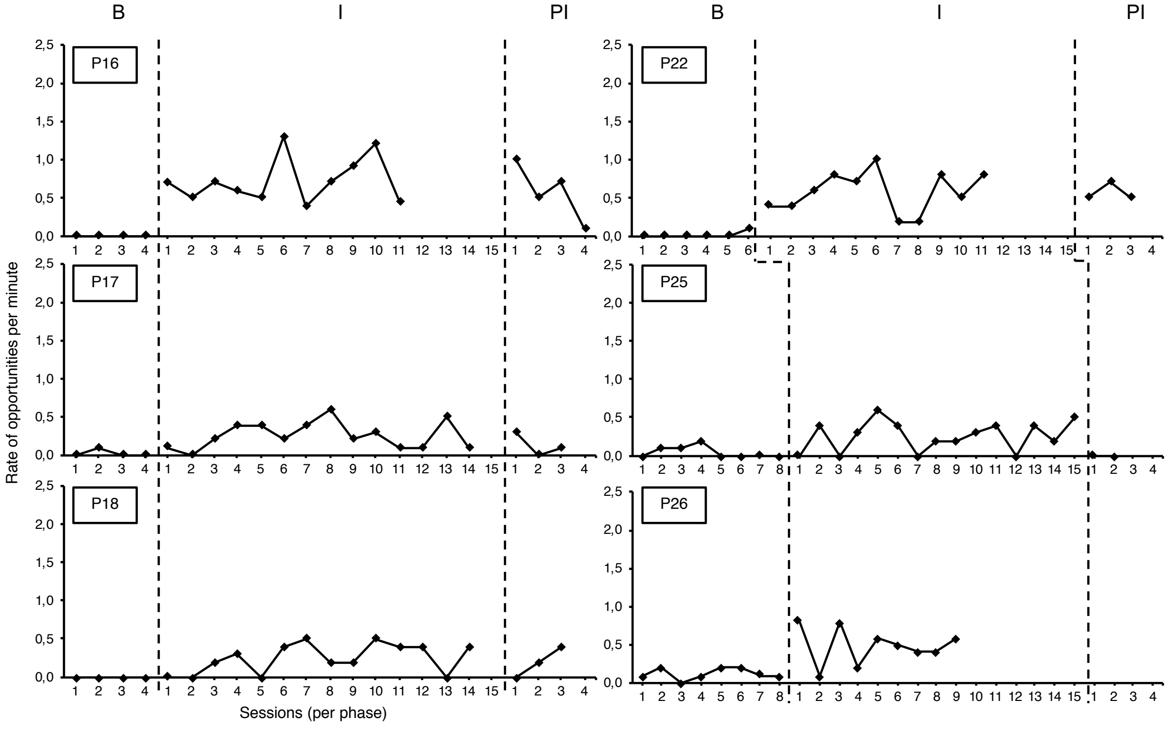

Figure 5. Rate of functional, early social, and empathic social initiations (individual parent education, treatment facility 1)

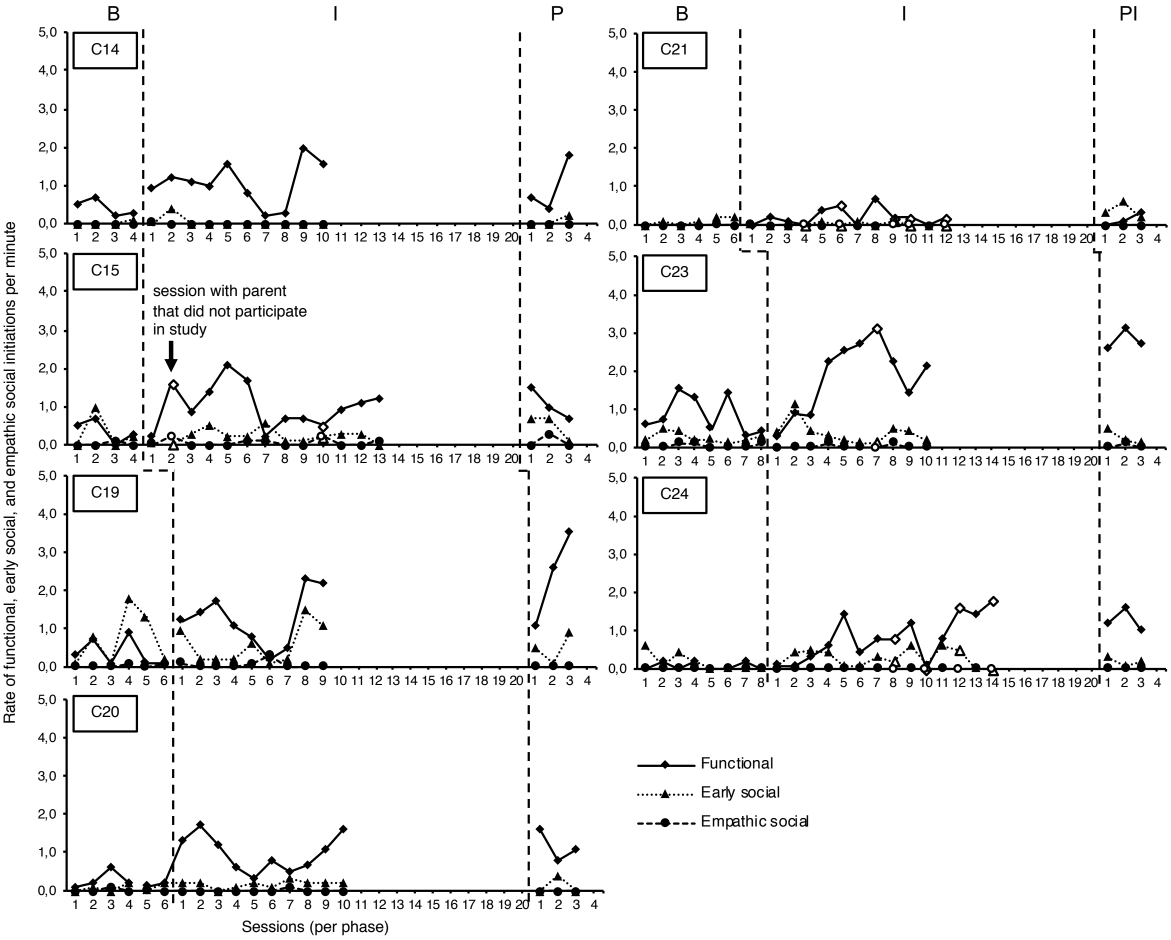

Figure 6. Rate of functional, early social, and empathic social initiations (individual parent education, treatment facility 2)

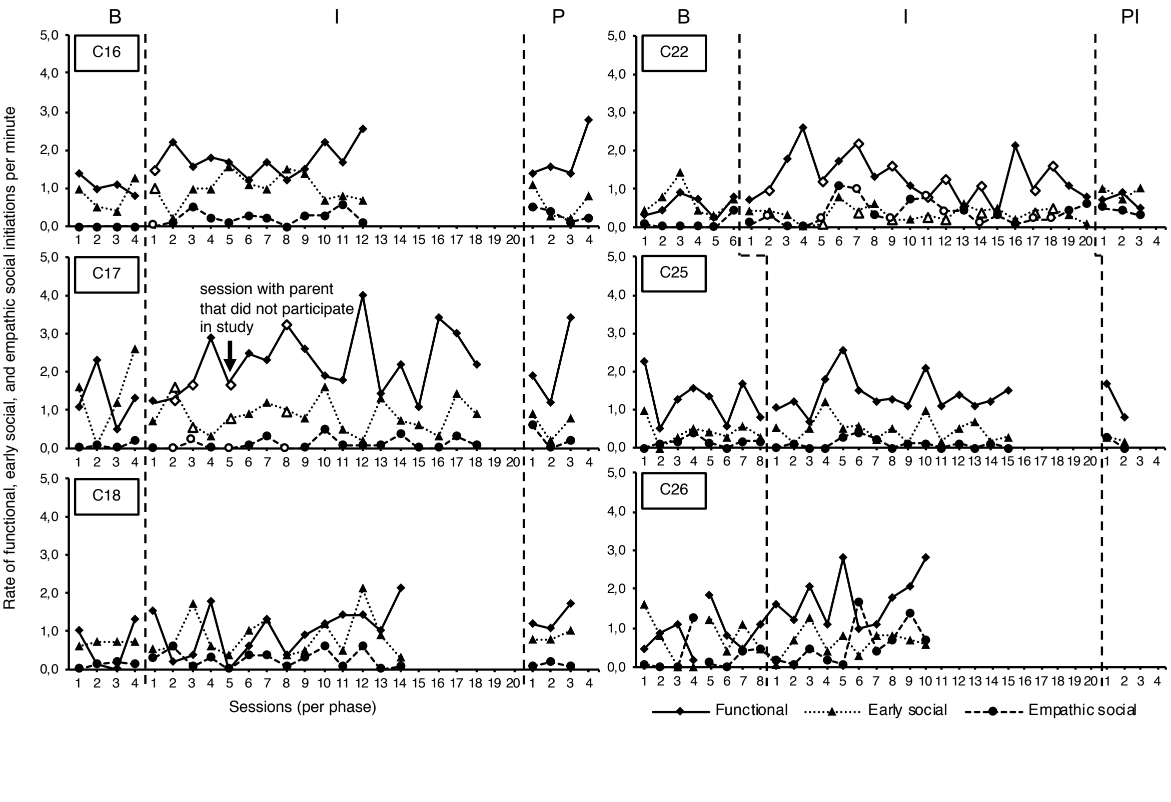

Online Resource 3. Tables providing median rates of parent-created opportunities and spontaneous child initiations during baseline, intervention, and post-interventions and values of Tau.

Table 1. Median rate of parent-created opportunities and values of Tau (group parent education)

	Median rate of opportunities			Tau	
Parent	Baseline	Intervention	Post-intervention	B-I contrast	I-PI contrast
1	0.00	0.00	0.00	.40	-.40
2	0.05	0.10	-	.13	-
3	0.03	0.20	-	.81*	-
4	0.00	0.10	0.40	.54[a]	.43
5	0.05	0.30	0.30	.46	-.14
6	0.00	0.30	0.30	.70*	-.22
7	0.10	0.10	0.10	.14	.14
8	0.10	0.20	-	.20	-
9	0.00	0.10	-	.64*	-
10	0.00	0.00	0.00	-.12	-.14
11	0.10	0.35	0.30	.94*	-.22
12	0.00	0.40	0.20	.64*	-.23
13	0.05	0.40	0.80	.83*	.73

Note. [a] Tau-U; *Significant at $\alpha = 0.05$.

Table 2. Median rates of functional and empathic social child initiations and values of Tau (group parent education)

Child	Median rate of functional initiations			Tau		Median rate of empathic social initiations			Tau	
	Baseline	Intervention	Post-intervention	B-I contrast	I-PI contrast	Baseline	Intervention	Post-intervention	B-I contrast	I-PI contrast
1	0.73	1.00	1.40	.33[a]	.53	0.00	0.00	0.20	.30	.56
2	0.85	0.90	-	-.04[a]	-	0.00	0.10	-	.50	-
3	1.00	1.80	-	.89*	-	0.25	0.30	-	.19[a]	-
4	0.30	0.80	1.10	.71*	.39	0.20	0.10	0.10	-.15	.17
5	0.40	0.80	1.40	.54[a]	.76	0.10	0.20	0.10	.57	-.38
6	0.60	1.90	1.25	.52[a]	-.22	0.45	0.56	1.00	.13	1.00*
7	0.70	1.15	0.90	.83*	-.50	0.10	0.25	0.30	.27	-.08
8	1.30	1.40	-	.35	-	0.15	0.30	-	.35	-
9	1.05	1.20	-	.24	-	0.00	0.00	-	.33	-
10	0.40	0.90	0.70	.42	-.26	0.10	0.10	0.10	.10	.28
11	1.50	1.61	0.70	.14	-.33	0.75	0.30	0.90	-.35	.60
12	0.50	0.80	0.90	.14	.13	0.25	0.50	1.00	.33	.50
13	1.50	1.50	2.60	.06[a]	.60	0.05	0.60	0.70	.91*	-.07

Note. [a] Tau-U; *Significant at $\alpha = 0.05$.

Table 3. Median rate of parent-created opportunities and values of Tau (individual parent education)

	Median rate of opportunities			Tau	
Parent	Baseline	Intervention	Post-intervention	B-I contrast	I-PI contrast
14	0.35	1.25	0.50	.90*	-.70
15	0.00	0.22	0.20	.45	-.13
16	0.00	0.70	0.60	1.00*	-.16
17	0.00	0.20	0.10	.83*	-.42
18	0.00	0.25	0.20	.71*	-.17
19	0.00	0.50	1.30	.56	.56
20	0.00	0.25	0.30	.90*	.07
21	0.00	0.17	0.50	.85*	.33
22	0.00	0.60	0.50	1.00*	-.06
23	0.00	0.54	1.10	.96*	.93*
24	0.00	0.25	0.30	.80*	.40
25	0.00	0.30	-	.61*	-
26	0.10	0.50	-	.82*	-

Note. [a] Tau-U; *Significant at $\alpha = 0.05$.

Table 4. Median rates of functional and empathic social child initiations and values of Tau (individual parent education)

	Median rate of functional initiations			Tau		Median rate of empathic social initiations			Tau	
Child	Baseline	Intervention	Post-intervention	B-I contrast	I-PI contrast	Baseline	Intervention	Post-intervention	B-I contrast	I-PI contrast
14	0.40	1.10	0.70	.70*	-.13	0.00	0.00	0.00	.00	.00
15	0.40	0.90	1.00	.67*	.08	0.00	0.00	0.00	.17	.08
16	1.05	1.70	1.50	.92*	-.15	0.00	0.20	0.30	.83*	.23
17	1.20	2.20	1.90	.63	-.06	0.05	0.10	0.20	.11	.31
18	0.56	1.10	1.20	.43	.27	0.13	0.30	0.10	.32	-.33
19	0.20	1.20	2.60	.78*	.59	0.00	0.00	0.00	.04	-.33
20	0.20	0.95	1.10	.92*	.23	0.00	0.00	0.00	-.07	-.10
21	0.00	0.20	0.10	.67*	-.14	0.00	0.00	0.00	.00	.00
22	0.55	1.15	0.70	.76[a]*	-.70	0.00	0.30	0.40	.65*	.30
23	0.65	2.15	2.70	.63*	.70	0.00	0.00	0.00	-.18	.13
24	0.00	0.80	1.20	.79*	.48	0.00	0.00	0.00	.00	.00
25	1.30	1.25	-	.07	-	0.15	010	-	-.28	-
26	0.85	1.70	-	.71[a]*	-	0.10	0.45	-	.40[a]	-

Note. [a] Tau-U; *Significant at α = 0.05.
